# Failing to Make Ends Meet: The Broad Clinical Spectrum of DNA Ligase IV Deficiency. Case Series and Review of the Literature

**DOI:** 10.3389/fped.2018.00426

**Published:** 2019-01-21

**Authors:** Aidé Tamara Staines Boone, Ivan K. Chinn, Carmen Alaez-Versón, Marco A. Yamazaki-Nakashimada, Karol Carrillo-Sánchez, María de la Luz Hortensia García-Cruz, M. Cecilia Poli, M. Edith González Serrano, Edgar A. Medina Torres, David Muzquiz Zermeño, Lisa R. Forbes, Francisco J. Espinosa-Rosales, Sara E. Espinosa-Padilla, Jordan S. Orange, Saul Oswaldo Lugo Reyes

**Affiliations:** ^1^Immunology Department at Hospital de Especialidades, UMAE 25 IMSS, Monterrey, Mexico; ^2^Center for Human Immunobiology, Texas Children's Hospital, Houston, TX, United States; ^3^Department of Pediatrics, Baylor College of Medicine, Houston, TX, United States; ^4^Genomic Diagnostic Laboratory at the National Institute for Genomic Medicine (INMEGEN), Mexico City, Mexico; ^5^Clinical Immunology Department, National Institute of Pediatrics, Mexico City, Mexico; ^6^Otolaryngology Department at the National Institute of Respiratory Diseases (INER), Mexico City, Mexico; ^7^Universidad del Desarrollo, Clínica Alemana de Santiago, Santiago de Chile, Chile; ^8^Immunodeficiencies Research Unit at the National Institute of Pediatrics (INP), Mexico City, Mexico; ^9^Department of Pediatrics, Vagelos College of Physicians and Surgeons, Columbia University, New York, NY, United States; ^10^Mexican Foundation for Girls and Boys with Primary Immunodeficiencies (FUMENI, AC), Huixquilucan, Mexico

**Keywords:** DNA repair defects, ligase IV deficiency, primary immunodeficiency, inborn error of immunity, case series, clinical spectrum

## Abstract

DNA repair defects are inborn errors of immunity that result in increased apoptosis and oncogenesis. DNA Ligase 4-deficient patients suffer from a wide range of clinical manifestations since early in life, including: microcephaly, dysmorphic facial features, growth failure, developmental delay, mental retardation; hip dysplasia, and other skeletal malformations; as well as a severe combined immunodeficiency, radiosensitivity, and progressive bone marrow failure; or, they may present later in life with hematological neoplasias that respond catastrophically to chemo- and radiotherapy; or, they could be asymptomatic. We describe the clinical, laboratory, and genetic features of five Mexican patients with LIG4 deficiency, together with a review of 36 other patients available in PubMed Medline. Four out of five of our patients are dead from lymphoma or bone marrow failure, with severe infection and massive bleeding; the fifth patient is asymptomatic despite a persistent CD4+ lymphopenia. Most patients reported in the literature are microcephalic females with growth failure, sinopulmonary infections, hypogammaglobulinemia, very low B-cells, and radiosensitivity; while bone marrow failure and malignancy may develop at a later age. Dysmorphic facial features, congenital hip dysplasia, chronic liver disease, gradual pancytopenia, lymphoma or leukemia, thrombocytopenia, and gastrointestinal bleeding have been reported as well. Most mutations are compound heterozygous, and all of them are hypomorphic, with two common truncating mutations accounting for the majority of patients. Stem-cell transplantation after reduced intensity conditioning regimes may be curative.

## Introduction

Inborn errors of immunity are a group of over 350 individually rare congenital diseases that result from mutations in one or more genes coding for proteins of the immune system. DNA repair defects result from mutations in any of several genes coding for proteins in a complex nuclear machinery that detect and fix double strand DNA breaks. Every cell in our body is exposed to 10 to 50 DNA double strand breaks per day, from intracellular mechanisms (meiosis, isotype class switch, gene recombination) and extracellular insults (free radicals, ionizing radiation, drugs) (1). In lymphocytes, a crucial process is the generation of diversity in T-cell receptors and immunoglobulins through V(D)J somatic recombination, which starts with the introduction of double strand breaks in the genes by RAG1 and RAG2 (2). Defects in the DNA repair machinery proteins (ATM, RAD50, NHEJ1, Artemis, NBN, SBDS, LIG4, MRE11, POLE) result in increased apoptosis, and oncogenesis. Most patients with DNA repair defects share a syndrome of neurologic deficits, combined (T and B-cell) immunodeficiency, bone marrow failure and/or hematologic neoplasia (3).

DNA Ligase IV is a 911-aminoacid protein, a nuclear enzyme with a DNA binding domain and two BRCT motifs, involved in double strand DNA repair through the non-homologous end-joining (NHEJ) pathway, the major repair mechanism in mammalian cells, which consists of at least 5 proteins. Specifically, the LIG4/XRCC4 complex oversees the final ligation step of the process (making ends of the repaired strands meet). Knockout models of the gene *LIG4*, located in human chromosome 13 and consisting of a single long exon, are lethal to mouse embryos because of massive neuronal apoptosis (4); all known human mutations are thus hypomorphic and result in a wide clinical spectrum, ranging from normal to severely compromised immune system with microcephaly, growth failure, facial dysmorphism, mental retardation, hypogonadism, progressive bone marrow failure, and leukemia or lymphoma. The physiopathology at a cellular level includes mutagenesis, apoptosis and oncogenesis, despite some residual function of LIG4, and despite a redundant DNA repair machinery. The resulting immune defect might be a radiosensitive T-B-NK+ severe-combined immunodeficiency. Even with a clinically normal phenotype, chances are that lymphocytes will have a restricted receptor repertoire, and that hematopoietic cells be susceptible to ionizing radiation and other insults, with cumulative DNA damage leading to bone marrow failure and hematologic malignancies (5).

Here, we describe five cases of DNA Ligase 4 deficiency (MIM phenotype number **#606593**) in two unrelated families from Mexico, whose clinical presentations ranged from lymphoma and early death, to mild disease. We reviewed the literature to further document the varying clinical presentations.

Written informed consent was obtained from the parents of all patients, for the publication of this case series, and any potentially identifying information, including face pictures and family trees.

## Case Reports

### Family A

A one-year-old boy from Northern Mexico was referred to UMAE-25 to rule out primary immunodeficiency. The third of four siblings, he was born at term to non-consanguineous parents.

An elder brother had died of Non-Hodgkin lymphoma of the brain at age 18 months old. He received BCG vaccination at birth, in 2005. At age 4 months he was hospitalized for viral encephalitis, and at 12 months for pneumonia and sepsis, with positive Cytomegalovirus (CMV) viral load (2,020 copies/ml); he suffered recurrent upper respiratory, gastrointestinal, and urinary infections; he developed oral candidiasis and a perianal ulcer caused by *Pseudomonas spp*; laboratory workup showed anemia (Hb 8.13 g/dL) and thrombocytopenia (80,000 per microliter), with normal leukocyte numbers and serum immunoglobulin levels: IgG 760 mg/dL, IgM 49 mg/dL, IgA 83 mg/dL; flow cytometry of lymphocyte subsets reported low CD4+ (110 cells/μl) and CD8+ (307 cells/μl) T-cells, with 1982 B-cells (43%), and 130 NK cells (9%); HIV infection was ruled out. A bone marrow aspirate showed myeloid maturation arrest with dysplastic forms. He was admitted with neurologic decay, fever, and systemic inflammation shortly before his death. The autopsy confirmed a diffuse large B-cell Non-Hodgkin lymphoma. Immunohistochemical staining for Epstein-Barr virus (EBV) was not available.

A second sister died at age 9 months of lung lymphoma. She started at 4 months old, with three episodes of pneumonia and one of sepsis; she suffered from milk protein allergy, vulvovaginal candidiasis, and diaper area dermatitis; her laboratory workup found: transient peripheral lymphopenia, low T-cells subsets (CD4+ 8 to 403 cells/mm^3^, CD8+ 6 to 232/mm^3^) with normal B and NK cells; normal serum immunoglobulin levels (IgG 853mg/dL, IgM 41, IgA 64 mg/dL, IgE < 18 IU/ml); positive CMV viral load, *Acinetobacter baumanii* cultured from blood, *Enterococcus faecium* from urine, and *Enterobacter aerogenes* from bronchial aspirate. Cystic fibrosis, HIV infection, tuberculosis, and gastroesophageal reflux disease were ruled out; a nitroblue tetrazolium (NBT) reduction assay was normal at 81%. The chest X-ray showed a paravertebral mediastinal mass; a chest computed tomography (CT) confirmed a well-delimited, right retrocardiac rounded mass shortly before her death. She deteriorated abruptly with metabolic acidosis, progressive respiratory distress and heart failure; she was admitted to the intensive care unit and received mechanical ventilation support, broad-spectrum antibiotic, milrinone, and cyclophosphamide, without improvement. The autopsy confirmed a diffuse large B-cell lymphoma; EBV staining was not performed.

Our patient, the third sibling, received the BCG vaccine at birth, without complications. Before 1 year of age he was treated for uncomplicated pharyngitis and avascular necrosis of the femoral head (Legg-Calvé-Perthes disease). Given his family history, he was started on oral trimethoprim/sulfamethoxazole (TMP/SMZ) and sent to our hospital for evaluation.

Other than an initially positive CMV viral load (440 copies/ml, age 1 year, treated with ganciclovir for 90 days), his first laboratory workup was unremarkable. A CT scan from head to abdomen was normal. Serum immunoglobulin levels and absolute lymphocyte counts were normal at age 1, 4, and 7 years old. In contrast, the CD4+ T-cell subset count has remained steadily low (163–657 cells/mm^3^, or 7–23%). The Mantoux tuberculin skin test was reactive at 11 mm of induration, at age 7 months. However, CFSE lymphocyte proliferation assay was low at age 9 months, and absent 3 years later, under: PMA/ionomycin, phytohemagglutinin, concanavalin, and anti-CD3/CD28 stimuli (Figure 3).

Whole-exome sequencing, performed at Texas Children's Hospital, revealed a compound heterozygous pathogenic variant (missense and small 5bp deletion) in *LIG4*: c.833G>A (p.R278H)/c.1271_1275delAAAGA (p.K424RfsX20). The p.K424R variant encodes a truncated protein missing 482 aminoacids; its frequency is lower than 0.03% for all populations according to ExAc (exac.broadinstitute.org). The p.R278H variant is predicted to be deleterious by all: DANN, GERP, dbNSFP, FATHMM, LRT, MetaLR, MetaSVM, MutationAssessor, MutationTaster, and PROVEAN *in silico* predictors. Both variants have been previously reported in patients with LIG4 deficiency and predicted to be deleterious by both SIFT and PolyPhen (transcript NM_206937.1, LIG4base, www.ensembl.org).

**Figure 1:**
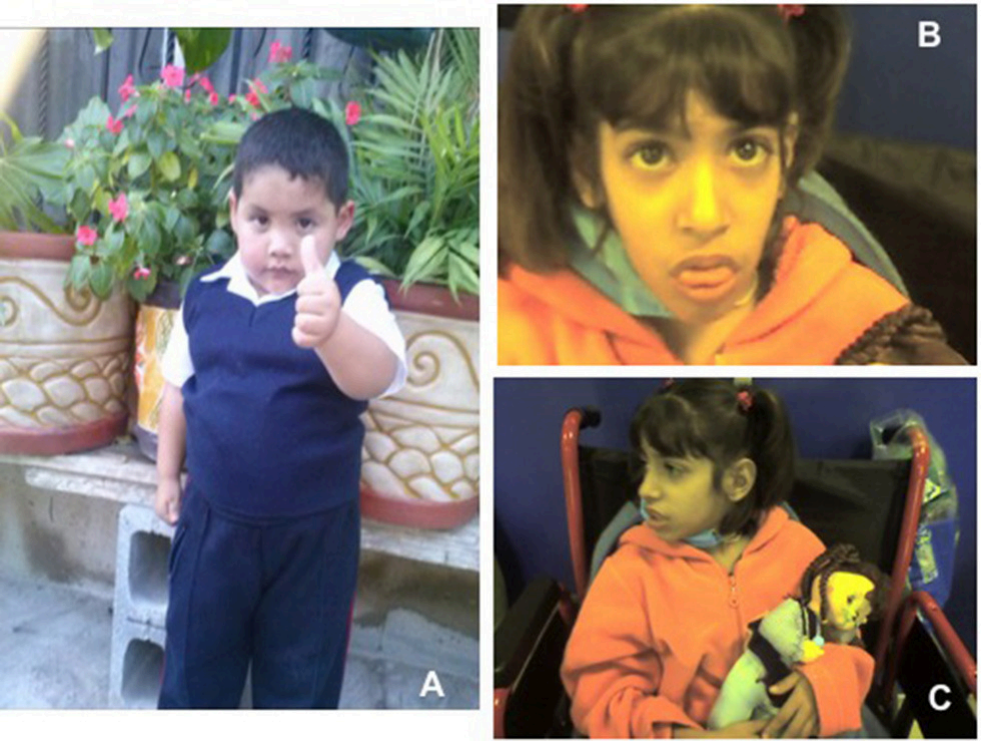
**(A)** Short stature with overweight in normocephalic school-age boy. Contrast with **(B,C)** Facial dysmorphisms seen in two twin sisters with LIG4 deficiency. Note prominent middle third of the face, long nose, micrognathia, long ears, jaundice, protruding tongue. (Face photos published with permission from their mothers).

**Figure 2:**
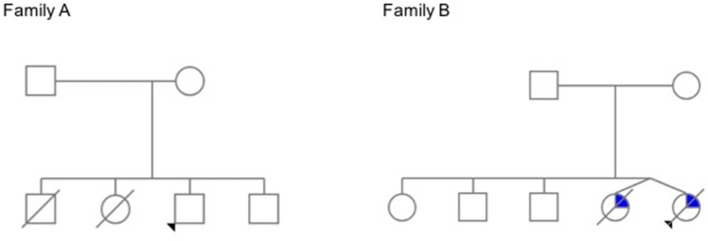
Family trees. In **Family A**, the elder siblings of the proband died young of lymphoma. The proband has a history of avascular necrosis of the femoral head, short stature and isolated CD4+ deficiency; his younger brother is asymptomatic. In **Family B**, teenage twin sisters died after a long history of recurrent infections, chronic liver disease, and progressive bone marrow failure (The pedigrees were built at pedigree.progenygenetics.com; they are included after written informed consent from the mothers).

In time, the patient, now 6 years old, developed obesity with normal stature and head circumference (see Figure 1), and no other manifestation. A younger brother, born in 2017, is also asymptomatic, in apparent good health (Figure 2).

**Figure 3:**
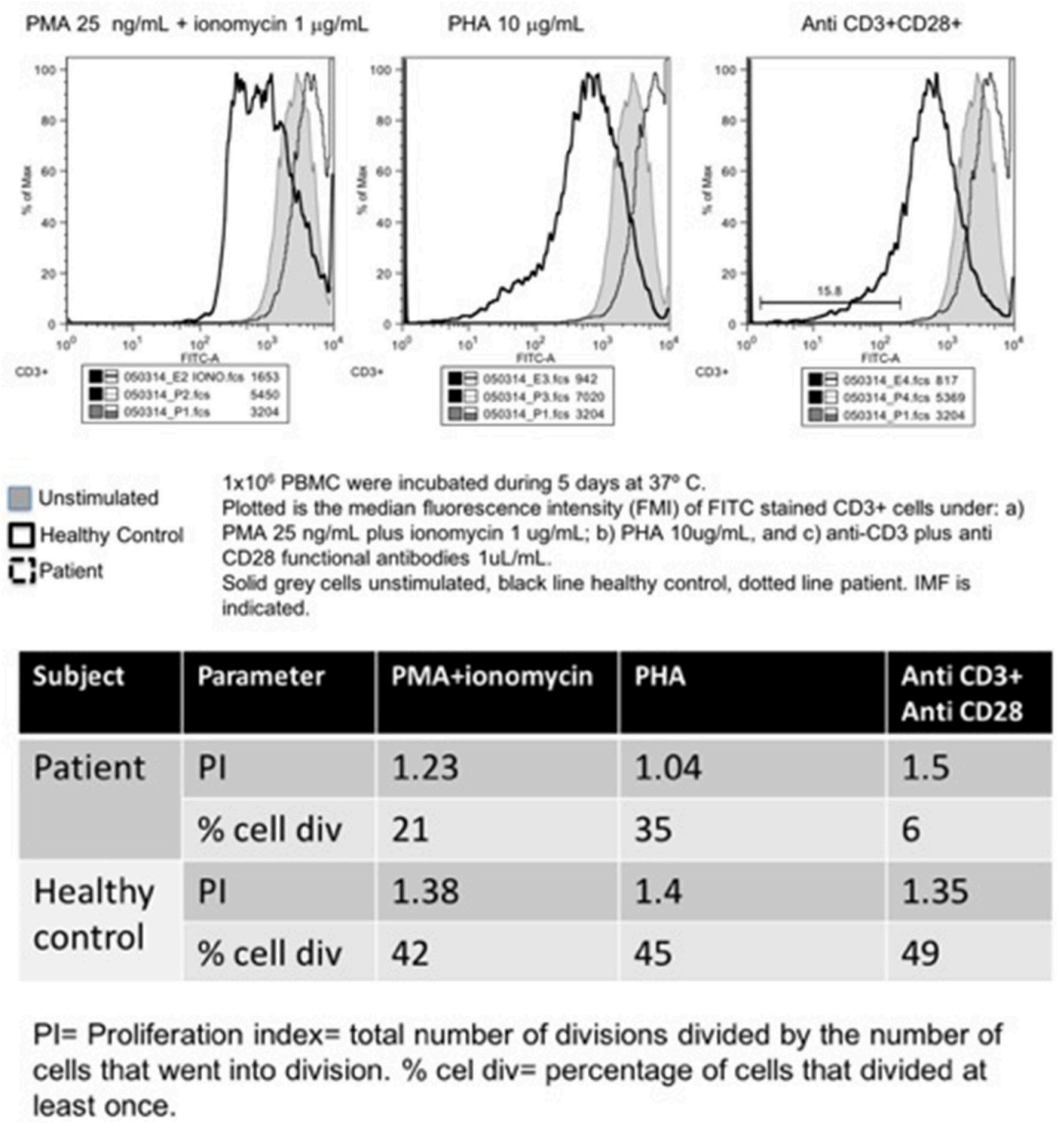
Carboxyfluorescein succinimidyl ester lymphoproliferation assay, plot (**upper panel**), and indexes (**lower panel**).

### Family B

Two teenage girl twins from Western Mexico were referred to INER for recurrent respiratory infections. They had a history of microcephaly and facial dysmorphism, with growth and psychomotor delay. Born pre-term (at 32 weeks of gestation) to non-consanguineous parents, the patients had three healthy elder siblings, and a pet dog.

During their first months of life, they suffered from 3 to 4 pneumoniae, gastroenteritis, and urinary tract infections. By 13 years of life, when they were referred to the National Institute of Pediatrics, they had developed moderate generalized jaundice with hepatosplenomegaly.

On physical examination, short stature (below percentile 10), low weight, mild to moderate mental retardation and speech delay were noted, with microcephaly below p3, a prominent mid-face with long nose, and micrognathia with a protruding tongue (see Figure 1). They had primary amenorrhea with absent secondary sexual features. A chest CT documented diffuse bilateral bronchiectasis; abdominal ultrasound and a barium swallow revealed portal hypertension with esophageal varices.

Laboratory workup showed persistent mild leukopenia with lymphopenia. Since around age twelve, they developed progressive pancytopenia: Hb 8g/dL, lymphocytes 210–300/mm^3^, less than 500 neutrophils, and 120,000 platelets; as well as elevated liver enzymes: increased conjugated bilirubin, ALT, AST, GGT, alkaline phosphatase, high ferritin (over 600 ng/ml), low fibrinogen (100 mg/dL), and high triglycerides (300 mg/dl). All serum immunoglobulins and lymphocyte subsets (CD3+, CD4+, CD8+, CD19+, CD16/56+) were also low: IgG 105–169 mg/dL, IgM 8.96–26.6 mg/dL, IgA < 6.67 mg/dL, IgE 1.1–1.5 IU/mL. A bone marrow aspirate reported medullary aplasia. Cellular radiosensitivity (radiomimetic assay) was negative to bleomycin; serologic tests for HepA, HepB, HepC, CMV, and EBV came back negative. Liver biopsy reported portal fibrosis with perivascular cell infiltration, suggestive of obstructive cirrhosis.

A provisional diagnosis of Nijmegen breakage syndrome (NBS) with bone marrow failure and sclerosing cholangitis was given. They continued treatment with prophylactic antibiotics and monthly intravenous gammaglobulin (IVIG), as well as ursodeoxycholic acid, molgramostin (GM-CSF), and oral transfer factor (leukocyte dialysate). They died 2 years apart at ages 14 and 16, at their hometown hospital, one twin of sudden massive gastrointestinal bleeding, and the other of sepsis and multiorgan failure (with pneumonia, anuria, and a distended abdomen). No autopsy was performed.

*NBN* was amplified and sequenced in Berlin with no mutations found. Years later, a targeted exome panel (Illumina TruSight) performed at INMEGEN in Mexico, revealed a compound heterozygous mutation in *LIG4*, consisting of the same 5 base-pair deletion as that of family A (c.1271_1275delAAAGA) in allele 1 (p. K424RfsX20), and a missense point mutation (c.745A>G) in allele 2 (p.M249V); both previously reported in patients with LIG4 deficiency (transcript NM_206937.1; LIG4base, www.ensembl.org), and predicted to be deleterious/probably damaging by both SIFT and PolyPhen. The p.M249V variant is absent from population databases such as gnomAD exomes; it is also predicted to be deleterious/probably damaging by all: DANN, GERP, dbNSFP, FATHMM, LRT, MetaLR, MetaSVM, MutationAssessor, MutationTaster, and PROVEAN *in silico* predictors.

## Review of the Literature

The first patient with LIG4 deficiency, a 14-year-old teenage boy with acute lymphoblastic leukemia who died 8 months after receiving prophylactic cranial radiotherapy, was characterized clinically in 1990 (6), and genetically in 1999 (7). Tables 1–3 summarize the clinical, laboratorial and genetic findings of all published patients available in PubMed Medline (https://www.ncbi.nlm.nih.gov/pubmed/) as of August 2018. Clinical and immunological manifestations vary widely, even among siblings with the same mutation. Table 4 summarizes the prevalence of neurological, developmental, infectious, and hematological components of LIG4 deficiency. The degree of protein truncation (early, mid, and late) seems to correlate with clinical, hematological, and immunological severity (mild, moderate, and severe).

**Table 1:** Demographic and clinical features of published patients with LIG4 deficiency.

**Patient**	**Origin**	**Sex**	**Family**	**Age at dx (years)**	**Onset**	**Clinical features**	**Status**	**References**
1 (180BR)	Turkish-cypriot	Male		14	B-symptoms	Weight loss, anorexia, lethargy, pallor, lymphadenopathy, hepatosplenomegaly: Acute lymphoblastic leukemia	Died 8 months after radiotherapy	6, 7
2	USA	Fem		26	Microcephaly and microsomy, lethargy and dizziness	Seckel syndrome. Cough, fever, pallor.	Died of pneumonia 2 months after chemotherapy	(8)
3 (411BR)	Germany	Male		9	Microcephaly	Dysmorphism, developmental delay and plantar warts.	Died at age 23 of lymphoma	(4, 9, 10)
4 (2303)	USA	Male	Sibling to 2304	46	Microcephaly	Chronic skin conditions, photosensitivity, telangiectasia, sinusitis	NR	(9)
5 (2304)	USA	Fem	Sibling to 2303	48	Microcephaly	Dysmorphism, growth failure, respiratory infections, psoriasis	NR	(9)
6 (99P0149)	Germany	Fem	Non-consang.	9	Microcephaly	Dysmorphism, dwarfism, mental delay, psoriasis	Alive and well 5 years after RIC HSCT from HLA-identical brother	(9, 11)
7 (3703)	Canada	Male	First cousin died of brain tumor in early childhood	4.75	Acute leukemia	Microcephaly, dysmorphism, developmental delay, hypogonadism, leukemia	Died shortly after chemotherapy	(12)
8 (SC2)	Turkish	Fem	Consang., heterozygous	1.5	Respiratory infections	Respiratory infections, candidiasis, chronic diarrhea, failure to thrive, fever	Died after conditioning, possibly due to veno-occlusive disease.	(13)
9 (P2)	German	Fem	Sister to 10	0	Microcephaly		Alive after HSCT	(14)
10 (P1)	German	Fem	Sister to 9	2	Recurrent infections	Chronic diarrhea, recurrent infections, failure to thrive, autoimmune cytopenia	Died shortly after chemotherapy	(14)
11 (P-1)	Morocco	Fem	Sister to 12	1.5	Repeated infections since 3 months old	Microcephaly, otitis, bronchiolitis, pneumonia and sepsis	Died of EBV PTLD 50 days after HSCT	(2)
12 (P-2)	Morocco	Fem	Sister to 11	0	Microcephaly		Died of VOD 2 months after HSCT	(2)
13	Japan	Fem		14	Mouth tumor, fever	Microcephaly, short stature, polydactyly,	Died after chemotherapy	(15)
14	Canada	Fem	Non-consang.	0.1	Omenn syndrome	Microcephaly, low weight, rash, hepatosplenomegaly, lymphadenopathy, diarrhea.	Alive and well 3.5 y after full conditioning HSCT	(16)
15	Turkish	Fem	Consang., sister to 16	10	Microcephaly, respiratory infections	Sinopulmonary recurrent infections	Alive and well after 2 RIC HSCTs	(8)
16	Turkish	Male	Consang., brother to 15	6	Ecchymoses	Low weight, upper respiratory and urinary infections	Alive	(8)
17 (F10)	Netherlands	Male		0.25	Microcephaly, dysmaturity, dysmorphism	Feeding difficulties, diarrhea, failure to thrive, icterus, tubulopathy, erythema	Died at 6 months of sepsis, respiratory insufficiency and severe gastrointestinal bleeding	(17, 18)
18	USA	Fem		34	Dubowitz syndrome	Rectorrhagia	Died of metastatic anal cancer	(18, 19)
19 (F1.1)	Canada	Fem	Sister to 20	17.5	Microcephaly	Small cerebral aneurysm, primary ovarian failure	Alive	(18)
20 (F1.2)	Canada	Fem	Sister to 19	11.75	Microcephaly	Atrial-ventricular septal defect, atrofphic kidney, rib hypoplasia, fusion of carpal bones, copper beaten skull, platybasia, abnormal C1 vertebrae, primary ovarian failure.	Alive	(18)
21 (F2)	USA	Fem		7.8	Microcephaly, malformations	Anal atresia with rectovaginal fistula, esotropia.	Alive	(18)
22 (F3)	Australia	Fem		2.1	Microcephaly, hip dysplasia	Unilateral congenital hip dysplasia, cutis marmorata.	Alive. BMT	(18)
23 (F4)	UK	Fem		2.5	Microcephaly, psoriasis	Psoriasis	Alive	(18)
24 (F5)	USA	Male		2	Microcephaly, hip dysplasia	Unilateral congenital hip dysplasia.	Alive	(18)
25 (F6)	Germany	Fem		2	Microcephaly, hip dysplasia	Congenital hip dysplasia, 2/3 toe syndactyly, excessive vomiting	Alive	(18)
26 (F7)	USA	Fem		3.67	Microcephaly, growth failure	None	Alive	(18)
27 (F8)	UK	Fem		1.75	Microcephaly, growth failure	None	Alive	(18)
28 (F9)	Turkey	Male		5.5	Microcephaly, hypopigmentation	Hypopigmentation, hypermobile knees, single palmar crease, 2/3 toe syndactyly, sandal gap	Alive	(18)
29 (P2)	North America	Fem	Sibling to P1 and P3	13	Recurrent pneumonias and sinusitis	Recurrent respiratory infections, microcephaly, short stature.	Alive	(5)
30(P1)	North America	Male	Sibling to P2 and P3	NA	NA	One walking pneumonia	Alive	(5)
31 (P3)	North America	Fem	Sibling to P1 and P2	NA	NA	Asymptomatic	Alive	(5)
32	Belarus	Fem		2	Stomatitis	Ulcerated stomatitis, encephalitis, lung and brain lymphoma.	Died of lymphoma	(20)
33 (C1)	African/Asian descent	Fem		7	Dysmorphic features, developmental delay	Respiratory infections, pancytopenia	Alive and well after HSCT	(21)
34 (C2)	Morocco	Male	Elder sister died at 18 mo. with pancytopenia	9	Chronic diarrhea	Chronic diarrhea, recurrent respiratory infections, hemorrhagic syndrome	Died of aplasia at age 10	(21)
35	Syria	Fem	Consang.	7	Dysmorphic features and developmental delay	Recurrent sinopulmonary infections, urosepsis, urofacial syndrome	Died of pneumonia	(22)
36	Italy	Male		6	Complicated pneumonia	Recurrent upper respiratory infections since age 1, dysgammaglobulinemia	Alive	(23)
37 LRL	Mexico	Fem	Two brothers. 3 first cousins died of infections before age 2	0.75	Pneumonia at 4 months		Died of lymphoma	This report
38 PRL	Mexico	Male	Two siblings	1.5	Pneumonia at 12 months		Died of lymphoma	This report
39 FRL	Mexico	Male	Two siblings	1	Pharyngoamigdal. at 2 months	Avascular necrosis of femoral head, overweight.	Alive and well	This report
40 MGP	Mexico	Fem	Twin sister	10	Recurrent respiratory infections	Microcephaly, dysmorphism, developmental delay, recurrent infections, jaundice	Died	This report
41 BGP	Mexico	Fem	Twin sister	10	Recurrent respiratory infections	Microcephaly, dysmorphism, developmental delay, recurrent infections, jaundice	Died	This report

*NR, Not reported*.

**Table 2:** Key features of published patients with LIG4 deficiency.

**Patient**	**Micro-cephaly**	**Dysmorphism**	**Growth failure**	**Developmental delay**	**Malignancy**	**Infections**	**Bone marrow failure**	**References**
1 (180BR)	No	No	No	No	AL Leukemia	None	No	(6, 7)
2	Yes	Receding forehead, hypertelorism, beaked nose, low set ears, micrognathia, downward palpebral fissures.	Yes	Mental retardation, high pitched voice.	AM Leukemia	Pneumonia (post chemo)	Yes (after chemo-therapy)	(8)
3 (411BR)	Yes	Bird-like	No	Yes, global	Lymphoma (Diffuse large B-cell, nasopharyngeal)	Extensive plantar warts	Pancytopenia	(4, 9, 10)
4 (2303)	Yes	Seckel-like	Yes	No	Myelodysplasia	Sinusitis	Pancytopenia	(9)
5 (2304)	Yes	Seckel-like	Yes	No	None	Chronic respiratory infections	No	(9)
6 (99P0149)	Yes	Seckel-like, Bird-like	Yes	Developmental/mental	None	Sinopulmonary recurrent	Pancytopenia, hypoplastic marrow	(9, 11)
7 (3703)	Yes, brachy.	NBS-like	No	Yes, significant cognitive delay	AL Leukemia	Neutropenic sepsis after chemo.	Aplasia after chemo.	(12)
8 (SC2)	No	No	No	No	None	Recurrent severe respiratory infections, candidiasis in diaper region, chronic diarrhea	Anemia, leukopenia	(13)
9 (P2)	Yes	No	No	Yes, significant neurodevelopmental	None		Lymphopenia	(14)
10 (P1)	Yes	No	No	No	Brain and lung non-Hodgkin B cell lymphoma	Sepsis, diarrhea	Neutropenia, lymphopenia, thrombocytopenia	(14)
11 (P-1)	Yes	No	Yes	No	None	Otitis, bronchiolitis, pneumonia, sepsis	Lymphopenia	(2)
12 (P-2)	Yes	No	No	No	None	None	Lymphopenia	(2)
13	Yes	No	Yes	No	Lymphoma (B-cell, NH) in upper gingiva and hard palate	Pulmonary aspergillosis	Progressive	(15)
14	Yes	No	Yes	Yes	None	None	Marked eosinophilia, mild lymphopenia	(16)
15	Yes	Low hairline, prominent nasal bridge, bilateral epicanthi	Yes	No	None	Sinopulmonary, ear.	Pancytopenia, progressive	(24)
16	Yes	Prominent nasal bridge, bilateral epicanthi	Yes	No	None		No	(24)
17 (F10)	Yes	Hypotelorism, small viscerocranium, flat philtrum, thin upper lip, preaxial polydactyly, brachimesophalangy, partial syndactyly	Yes	Neurologic abnormalities	None	Urinary tract, sepsis	Thrombocytopenia	(17, 18)
18	Yes	Yes	NR	No	Anal cancer	None	Pancytopenia	(18, 19)
19 (F1.1)	Yes	No	Yes	Yes, mild	None	Mild. Influenza	Pancytopenia, self-resolved. Thrombocytopenia	(18)
20 (F1.2)	Yes	Malformations	Yes	Yes, mild-moderate	None	Mild recurrent (respiratory, skin, GI)	Pancytopenia, persistent. Thrombocytopenia	(18)
21 (F2)	Yes	Malformation	Yes	Yes, mild	None	None	Pancytopenia, self-resolved. Thrombocytopenia	(18)
22 (F3)	Yes	No	Yes	Yes, mild	None	Mild recurrent (respiratory, skin, GI)	Thrombocytopenia	(18)
23 (F4)	Yes	No	Yes	No	None	None	Anemia, thrombocytopenia	(18)
24 (F5)	Yes	No	Yes	No	None	None	Lymphopenia	(18)
25 (F6)	Yes	Malformations	Yes	Yes, mild	None	Mild recurrent (respiratory, skin, GI)	Thrombocytopenia	(18)
26 (F7)	Yes	No	Yes	No	None	None	Thrombocytopenia	(18)
27 (F8)	Yes	No	Yes	Yes, mild	None	None	Thrombocytopenia	(18)
28 (F9)	Yes	Malformations	Yes	Yes, mild	None	Mild recurrent (respiratory, skin, GI)	Thrombocytopenia	(18)
29 (P2)	Yes	No	Yes	No	None	Pneumonia and sinusitis	Lymphopenia	(5)
30(P1)	unknown	No	Small stature		None	Walking pneumonia	Mild neutropenia	(5)
31 (P3)	No	No	No		None	None	Lymphopenia, mild neutropenia	(5)
32	No	No	No	No	Lymphoma, EBV-pos; diffuse large B-cell; lung and brain	EBV stomatitis/encephalitis	No	(20)
33 (C1)	Yes	Yes	Yes	Yes, mild	None	Respiratory	Slowly progressive pancytopenia	(21)
34 (C2)	Yes	Yes	Yes	Severe language delay, moderate mental retardation	None	Respiratory, warts	Yes	(21)
35	Yes	Yes	Yes	Yes, mild		Pneumonia, otitis media, sinusitis, oral candidiasis. Urosepsis	Pancytopenia	(22)
36	No	No	No	No	None	Sinopulmonary recurrent, ear	No. Progressive lymphopenia	(23)
37 LRL	No	No	No	No	Lung lymphoma	Pneumonia 3x	No	This report
38 PRL	No	No	No	No	Brain B-cell lymphoma (Non-Hodgkin)	Pneumonia, Perianal ulcers, Sepsis	No	This report
39 FRL	No	No	Yes	No	None	Mild upper respiratory.	No	This report
40 MGP	Yes	Yes	Yes	Yes	None	Recurrent: sinopulmonary, gastrointestinal, urinary	Yes	This report
41 BGP	Yes	Yes	Yes	Yes	None	Recurrent: sinopulmonary, gastrointestinal, urinary	Yes	This report

*NR, Not reported*.

**Table 3:** Immunological and genetic features of published patients with LIG4 deficiency.

**Pat**	**Germs (isolates)**	**Serum immunoglobulins**	**Other (comment)**	**Mutation type**	**Allele 1**	**Allele 2**	**References**
1 (180BR)	NR	Not reported	Prophylactic cranial radiation: ulcers, lethargy, tetraparesis	hom, miss	c. 833 G>A (p. R278H)	c. 833 G>A (p. R278H)	(6, 7)
2	NR	Not reported	Chromosomal abberrations. Chemotherapy: severe toxicity	NR	ND	ND	(8)
3 (411BR)	NR	Low	Microcephaly at birth, not evident at age 9. Null B-cells, low T. T-B-NK+ CID	hom, miss, (+2 polymorphisms)	c. 833 G>A/8 C>T/26 C>T (p. R278H/A3V/T9I)	c. 833 G>A/8 C>T/26 C>T (p. R278H/A3V/T9I)	(4, 9, 10)
4 (2303)	NR	Not reported	Hypothyroidism, type 2 diabetes, hypogonadism	comp het, nons	c. 1738 C>T (p.R580X)	c. 2440C>T (p.R814X)	(9)
5 (2304)	NR	Not reported	Hypothyroidism, amenorrhea	comp het, nons	c. 1738 C>T (p.R580X)	c. 2440C>T (p.R814X)	(9)
6 (99P0149)	NR	All normal	Multiple psoriasiform erythrodermic, squamous skin patches, atypical bone maturation, low CD3 and CD19	comp het, miss/nons	c. 1406 G>A (p.G469E)	c. 2440C>T (p.R814X)	(9, 11)
7 (3703)	NR	Not reported	Low birth weight, criptorchidism, hypogonadism, clinodactyly	hom, nons	c.2440C>T (p.R814X)	c. 2440C>T (p.R814X)	(12)
8 (SC2)	Candida	Low IgG, IgA and IgM	T-B-NK+ SCID, reduced lymphoproliferation (PHA, aCD3)	hom, small del	g.5333_5335delCAA (p. Q433del)	g.5333_5335delCAA (p. Q433del)	(13)
9 (P2)	No	Low IgM, absent IgA	T-B-NK+ SCID, isohemagglutinins present	comp het, miss/nons	c.1118A>T (p. H282L)	c.1544_1548delAAAGA (p.D423fs442X)	(14)
10 (P1)	S. pneumoniae, HHV6, Norwalk, EBV, Aspergillus	High IgG and IgM, normal IgA	T-B-NK+ SCID, small thymus, autoimmune thrombocytopenia, no tetanus, diphteria or pneumococcus titers	comp het, miss/nons	c.1118A>T (p. H282L)	c.1544_1548delAAAGA (p.D423fs442X)	(14)
11 (P-1)	S. pneumoniae	Absent IgA, Low IgG and IgM	T-B-NK+ SCID, reduced lymphoproliferation	comp het, miss/small del	c.1544_1548del5bp (p.K424fs20X)	c.1112A>G (p.Q280R)	(2)
12 (P-2)	No	Low IgM, absent IgA (maternal IgG)	T-B-NK+ SCID, normal lymphoproliferation	comp het, miss/small del	c.1544_1548del5bp (p.K424fs20X)	c.1112A>G (p.Q280R)	(2)
13	Aspergillosis after neutropenia	Low IgM and IgG	T-B-NK+ CID	comp het, miss/small del	c.745A>G (p.M249V)	c.1270_1274del5bp (p.K424fs20X)	(15)
14	EBV after HSCT	Low IgM and IgA	Low TRECs, B cells, lymphoproliferation, GVHD	comp het, miss/small del	c.845A>T(p.H282L);c.26C>T (SNP)	c.1747_1751del5bp (p.R581fsX)	(16)
15	NR	Low IgM	Spontaneous chromosomal breakages increased, normal bone marrow cellularity	hom, small del	c.1762delAAG (p.K588del)	c.1762delAAG (p.K588del)	(8)
16		Low IgG and IgM	Inguinal hernia, spontaneous chromosomal breakage, normal BM cellularity	hom, small del	c.1762delAAG (p.K588del)	c.1762delAAG (p.K588del)	(8)
17 (F10)	P aeruginosa, E faecalis, P jiroveci, rhinovirus, norovirus, astrovirus, C difficile, Candida	Low IgG and B-cells	Polydactyly, dysplastic kidneys, corpus callosum dysgenesia, very low B-cells	comp het, SN del trunc/trunc	c.613delT (p.S205LfsX29)	c. 1904delA (p.Lys635ArgfsX10)	(17, 18)
18	No	NR	Radiotherapy: desquamative skin injury	comp het, SN del trunc/nons	c.613delT (p.S205LfsX232)	c.2440C>T (p.R814X)	(18, 19)
19 (F1.1)	NR	Low IgG	Low CD4+, null CD19+	comp het, miss/small del	c.2440C>T (p.R814X)	c.1271_1275delAAAGA (p.K424RfsX20)	(18)
20 (F1.2)	NR	Low IgG	Low CD4+, null CD19+, very low naïve T cells	comp het, miss/small del	c.2440C>T (p.R814X)	c.1271_1275delAAAGA (p.K424RfsX20)	(18)
21 (F2)	NR	NR	NR	comp het, nons	c.2440C>T (p.R814X)	c.2094C>G (p.Y698X)	(18)
22 (F3)	NR	Low IgG	Low CD3, CD8, very low CD19	comp het, dup/nons	c.2440C>T (p.R814X)	c.2386_2389dupATTG (p.A797DfsX3)	(18)
23 (F4)	NR	Low IgG	Low CD4+, very low CD19+	comp het, nons/small del	c.2440C>T (p.R814X)	c.1271_1275delAAAGA (p.K424RfsX20)	(18)
24 (F5)	NR	Low IgG	Low CD3, CD4, CD8, very low CD19	comp het, nons/small del	c.2440C>T (p.R814X)	c.1271_1275delAAAGA (p.K424RfsX20)	(18)
25 (F6)	NR	Low IgG	Very low CD19+	comp het, nons/small del	c.2440C>T (p.R814X)	c.1271_1275delAAAGA (p.K424RfsX20)	(18)
26 (F7)	NR	NR	NR	comp het, nons/small del	c.2440C>T (p.R814X)	c.1512_1513delTC (p.R505CfsX12)	(18)
27 (F8)	NR	Low IgG	NR	comp het, nons/small del	c.2440C>T (p.R814X)	c.1246_1250dupGATGC (p.Leu418MetfsX3)	(18)
28 (F9)	NR	Normal	Very low CD19+	comp het, nons/small del	c.2440C>T (p.R814X)	c.1271_1275delAAAGA (p.K424RfsX20)	(18)
29 (P2)	NR	Panhypogammaglobulinemia	Low CD3+, CD19+, naïve T	comp het, miss/nons	c.2440C>T (p.R814X)	c.1345A>C (p.K449Q)	(5)
30(P1)	NR	Normal IgG	Low CD19+, naïve T cells	comp het, miss/nons	c.2440C>T (p.R814X)	c.1345A>C (p.K449Q)	(5)
31 (P3)	No	Low IgG	Low CD3+, CD19+, naïve T	comp het, miss/nons	c.2440C>T (p.R814X)	c.1345A>C (p.K449Q)	(5)
32	EBV	Low IgA	Low T cells	comp het, small del/miss	c.2736+3delC	c.8C>T (p.A3V); c.26C>T(p.T9I)	(20)
33 (C1)	NR	Low IgG and IgM		comp het, nons/small del	c.2440C>T (p.R814X)	c.1271_1275delAAAGA (p.K424RfsX20)	(21)
34 (C2)	HPV	NR		comp het, miss/small del	c.847A>G (p.K283E)	c.1271_1275delAAAGA (p.K424RfsX20)	(21)
35	S. pneumoniae, H. influenzae	Low IgG2 and IgM	Low CD4+, null CD19+. Urofacial syndrome with hom. LRIG2 mut.	hom, miss	c.T1312C (p.Y438H)	c.T1312C (p.Y438H)	(22)
36	NR	Absent IgA and IgM. Normal IgG, low IgG3	Very low T and B cells. Poor response to vaccines. Bronchiectasis	comp het, miss/small del	c. 833 G>A (p. R278H)	c.1271_1275delAAAGA (p.K424RfsX20)	(23)
37 LRL	Acinetobacter baumanii, Enterococcus faecium, Enterobacter aerogenes, CMV	All normal	Low T cells	NR	ND	ND	This report
38 PRL	Pseudomonas, CMV	All normal	Low T cells	NR	ND	ND	This report
39 FRL	No	All normal	Legg-Calvé-Perthes disease, right hip. Low CD4+, low lymphoproliferation.	comp het, miss	c.1236T>A (p.N412K)	c.32C>G (p.A11G)	This report
40 MGP	NR	Low IgG, IgA and IgM	Primary amenorrhea, sclerosing cholangitis	comp het, small del/miss	c.1271_1275delAAAGA (p.K424RfsX20)	c.745A>G (p.M249V)	This report
41 BGP	NR	Low IgG, IgA and IgM	Primary amenorrhea, sclerosing cholangitis	NR	ND	ND	This report

*ND, Not done; NR, Not reported; NT, Not tested; IR, ionizing radiation; BLM, bleomycin; DEB, diepoxybutane; MITC, mitomycin C. All reported mutations are hypomorphic*.

**Table 4:** Prevalence of clinical and laboratory features found in 41 LIG4 deficiency patients.

Female	28/41	68%
Microcephaly	32/40	80%
Dysmorphic facial features	15/41	37%
Growth failure	28/40	70%
Syndactyly/Polysindactyly	4/41	10%
Other malformations	3/41	7%
Congenital hip dysplasia	4/41	10%
Infections (any)	28/41	68%
Sinopulmonary infections	23/41	56%
Skin conditions	8/41	20%
Warts	2/41	5%
Hypogammaglobulinemia	25/30	83%
Very low CD19+ B-cells	24/30	80%
Malignancy	10/41	24%
Bone marrow failure	17/39	44%
Radiosensitivity	24/28	86%

*(100% radiosensitivity with ionizing radiation)*.

## Discussion

We describe a series of five LIG4 deficiency patients with a wide spectrum of clinical manifestations, ranging from short stature and asymptomatic CD4+ lymphopenia, to liver failure with sudden massive bleeding.

Although relatively small, this is one of the largest case series to date on the subject of LIG4 deficiency; the genetic diagnosis was confirmed in both families by whole-exome sequencing only, but we also reviewed the literature for all published patients to date (our job was aided by a recent review by Altmann and Gennery (1) comprising 28 LIG4 deficient patients up until 2016).

Forty-one LIG4 deficiency patients have been described to date (In addition, Bluteau et al. recently found three cases of LIG4 deficiency among their cohort of bone marrow failure patients, although their features were not described in detail) (25). Their phenotypes are varied, with more clinical severity associated to early mutations and a truncated protein. The degree of severity is probably linked to the amount of residual function of the ligase, but confounded by the complex interactions and the intricate redundancy of the DNA repair defect machinery.

To our knowledge, our patients from family B are the first to develop sclerosing cholangitis (SC) in the context of LIG4 deficiency. SC has been reported mainly in X-linked Hyper-IgM syndrome due to CD40L deficiency, but also in other forms of combined immunodeficiencies (26). This autoimmune complication is thought to result from the colonization of the bile ducts by Cryptosporidium and microsporidium from the small intestine, eventually leads to biliary cirrhosis and liver failure, which greatly complicates prognosis. SC should be considered in patients with lymphocyte defects and cholestatic jaundice. All such patients should be warned against drinking unpurified water, and against having close contact with dog pets.

When there is a full-blown phenotype of combined immunodeficiency, we can fear the complications of malignancy and bone marrow failure. In contrast, it is unclear what the best approach is with asymptomatic patients carrying a deleterious variant in *LIG4*. In theory, they will accumulate DNA double-strand breaks and cell damage that result in increased apoptosis of hematopoietic cells, with the consequent risk of late-onset aplastic anemia and malignancy. Felgentreff et al. (5) recently reported immunological findings in two asymptomatic siblings who shared a *LIG4* bi-allelic mutation (K449Q/R814X) with their sister (the proband), but not a history of infections; all three siblings with compound heterozygous mutations had: low mean corpuscular volume, B-cell lymphopenia, shortened telomeres, reduced naïve T-cells, and increased radiosensitivity, as well as some skewing of their lymphocytes' receptor repertoires. The immune defects are thus present but clinically silent, and environmental or infectious triggers might precipitate bone marrow failure or myelodysplasia.

The differential diagnosis of LIG4 deficiency includes Nijmegen Breakage Syndrome (NBS), RAD50 and NHEJ1 deficiencies; Fanconi anemia, and any other congenital disease presenting with: microcephaly, facial dysmorphisms (described as bird-like facies); hypogammaglobulinemia, and low B-cells; including IKAROS and NFKB2 deficiencies. Dubowitz and Seckel's are clinical syndromes of which LIG4 deficiency patients undoubtedly represent a considerable subset of cases (19). Of note, LIG4 deficient patients may first seek medical care for hematologic malignancies, but respond badly to chemotherapy and radiotherapy; the astute clinician should consider this diagnosis in patients with suggestive clinical features (12), and consider instead hematopoietic stem-cell transplantation (HSCT) after a reduced intensity conditioning (RIC) regime.

Not all patients have had microcephaly, but all had radiosensitivity when their cell cultures were exposed to ionizing gamma radiation. Our twin sisters' cells were reported normal or negative for the bleomycin (BLM) radiomimetic test, which has been described to have a strong positive correlation when compared to ionizing radiation with gamma rays. However, it has also been found to be more selective and less predictable than gamma radiation (27–29). BLM is an antibiotic obtained from *Streptomyces verticillus*, used in the treatment of cancer and considered a radiomimetic for its induction of single and double DNA strand breaks, in the presence of iron ions in cell cultures (27).

Given the dreaded fatal complications of hematologic neoplasia, bone marrow failure and chronic liver disease, the best chance LIG4-deficient patients with severe (hematological/immunological) phenotypes have of reaching adulthood, might be to receive an HSCT (11, 30) under the best possible conditions (HLA-identical donor, a reduced intensity conditioning regime with low-dose Cyclosporin A (24, 30, 31), infection-free, no organ failure). The neurological deficits and mental retardations are usually mild enough to allow for a happy and productive life in transplant survivors, capable of loving and being loved, and of learning a craft.

In conclusion, LIG4 deficient patients may present soon after birth or later in life, with, or without: a family history of consanguinity and affected siblings, microcephaly, beaked nose, T-B-NK+ SCID, Omenn syndrome, low serum immunoglobulins, and very low B-cells, worsening pancytopenia, radiosensitivity; and they may develop bone marrow failure, sepsis, severe bleeding, and/or hematologic malignancies. Early diagnosis is crucial, as hematologic and infectious complications are more frequent with increasing age, and because RIC HSCT may be curative.

## Ethics Statement

The molecular and genetic diagnostic studies were approved by the Institutional Review Board (Comité de Investigación) at the National Institute of Pediatrics. Said studies were preceded by informed consent from the patients' next of kin. Face photographs are included with authorization from their mothers.

## Author Contributions

AB coordinated the care of one patient, conceived the manuscript and helped collect data. IC supervised and performed the analysis of exome sequencing for one patient. CA-V supervised and analyzed exome sequencing for one patient, edited the manuscript, and participated in scientific discussions. MY-N coordinated the care of two patients, proposed differential diagnoses and discussed the manuscript. KC-S performed the WES for one of the patients. MG-C coordinated the care of two patients. MP participated in the genetic diagnosis of one patient, and discussed the manuscript. MG performed and analyzed lymphoproliferation assays. EM participated in the molecular and genetic diagnoses of one patient. DM participated in clinical data collection. LF was involved in the genetic diagnosis of one patient and reviewed the manuscript. FE-R facilitated the genetic diagnosis of one patient, and participated in the care of two patients. SE-P participated in the care, and coordinated the molecular and genetic diagnoses of one patient. JO coordinated the genetic diagnosis of one patient. SL conceived and wrote the manuscript, participated in the care of two patients, and built the tables. All the authors read and contributed to the final draft of the manuscript.

### Conflict of Interest Statement

The authors declare that the research was conducted in the absence of any commercial or financial relationships that could be construed as a potential conflict of interest.

## Abbreviations

ALT: alanine aminotransferase
AST: aspartate aminotransferase
BCG: Bacille Calmette-Guérin
BCM, Baylor College of Medicine: Houston
BLM: Bleomycin
BRCT: C-terminal domain of breast cancer susceptibility protein
CFSE: Carboxyfluorescein diacetate succinimidyl ester
CMV: cytomegalovirus
DNA: deoxyribonucleic acid
GGT: Gamma-glutamyl transferase
GM-CSF: granulocyte-macrophage colony stimulating factor
IEI: Inborn errors of immunity
INER, National Institute of Respiratory Diseases: Mexico
INP, National Institute of Pediatrics: Mexico
INMEGEN, National Institute of Genomic Medicine: Mexico
IVIG: intravenous immunoglobulin
LIG4: DNA Ligase 4
NBN: Nibrin
NBS: Nijmegen Breakage Syndrome
NHEJ: Non-homologous end join
PCR: Polymerase chain reaction
SC: sclerosing cholangitis
TMP/SMZ: trimethoprim/sulfamethoxazole
UMAE 25: Unidad Médica de Alta Especialidad No.
